# ChloS-HRM, a novel assay to identify chloramphenicol-susceptible *Escherichia coli* and *Klebsiella pneumoniae* in Malawi

**DOI:** 10.1093/jac/dky563

**Published:** 2019-01-25

**Authors:** Christopher T Williams, Patrick Musicha, Nicholas A Feasey, Emily R Adams, Thomas Edwards

**Affiliations:** 1 Research Centre for Drugs and Diagnostics, Liverpool School of Tropical Medicine, Liverpool, UK; 2 Malawi-Liverpool-Wellcome Trust Clinical Research Programme, Queen Elizabeth Central Hospital, Blantyre, Malawi; 3 Mahidol-Oxford Tropical Medicine Research Unit, Mahidol University, Bangkok, Thailand; 4 Centre for Tropical Medicine and Global Health, Nuffield Department of Medicine, University of Oxford, Oxford, UK; 5 Department of Clinical Sciences, Liverpool School of Tropical Medicine, Liverpool, UK

## Abstract

**Objectives:**

Chloramphenicol is a broad-spectrum antimicrobial widely available in sub-Saharan Africa. With susceptibility re-emerging among Enterobacteriaceae in Blantyre, Malawi, we designed and evaluated a new high-resolution melt (HRM) RT-PCR assay, ChloS-HRM, to identify chloramphenicol-susceptible infections in a hospital setting.

**Methods:**

Seventy-two previously whole-genome sequenced isolates of *Escherichia coli* and *Klebsiella pneumoniae* from the Queen Elizabeth Central Hospital, Malawi, were subjected to determination of chloramphenicol MICs. Primers were designed to detect 18 chloramphenicol resistance genes that produce seven distinct peaks correlating with different gene groups (*catA1, catA2, catA3, catB2, catB* group 3, *cmlA* and *floR*) following HRM analysis. ChloS-HRM results were compared with MIC and WGS results.

**Results:**

ChloS-HRM correctly identified 15 of 17 phenotypically susceptible isolates and 54 of 55 resistant isolates, giving an accuracy of 88% in identifying susceptibility and 98% in identifying resistance. WGS identified 16 of 17 susceptible and 54 of 55 resistant isolates, giving an accuracy of 94% in identifying susceptibility and 98% in identifying resistance. The single false-susceptible result had no detectable gene by ChloS-HRM or WGS. Compared with WGS, ChloS-HRM had 100% sensitivity and specificity for *catA* (*catA1–3*), *cmlA* and *floR*, and 96% specificity for *catB*; sensitivity could not be estimated due to the lack of *catB* in the clinical sample collection. The overall agreement between MIC and HRM was 96% and between MIC and WGS it was 97%.

**Conclusions:**

ChloS-HRM could support antimicrobial stewardship in enabling de-escalation from third-generation cephalosporins by identifying chloramphenicol-susceptible infections. This would be valuable in areas with chloramphenicol-susceptible MDR and XDR Enterobacteriaceae.

## Introduction

The greatest burden of antimicrobial resistance (AMR) is in the developing world where severe bacterial infection is an important cause of morbidity and mortality^1^ and third-generation cephalosporins are frequently both first- and last-line antimicrobials.

Although AMR prevalence data from sub-Saharan Africa are limited, available data consistently reveal that AMR is increasing, particularly in the Enterobacteriaceae,^2^ with resistance to third-generation cephalosporins encountered in up to 46% of isolates depending on the setting.^3^ Between 1996 and 2016 in Malawi 68.3% of Gram-negative infections were resistant to first-line drugs amoxicillin or penicillin, chloramphenicol and co-trimoxazole compared with just 6.6% of Gram-positive infections.^4^

With the current paucity of new drugs in the pipeline,^5^ reimplementing older ‘forgotten’ drugs is a potential solution, particularly in resource-limited settings.^6^ Many of these drugs, such as chloramphenicol, were introduced in the 1940s–70s and increasing resistance has led to their restriction or removal from clinical use.^7^ In their absence susceptibility has returned, increasing their utility in treating susceptible infections.^8^^,^^9^ This is particularly important with the increasing prevalence of ESBLs and carbapenemases reducing the effectiveness of first-line agents.

Chloramphenicol was discovered in 1947^10^ and became widely used due its broad activity against many Gram-positive and -negative species; however, by the 1960s it had largely been abandoned in high-income countries due to its toxicity profile.^11^ Despite this it remains a key drug in low- and middle-income countries due to its low price and ease of production.

Reports of returning chloramphenicol susceptibility are increasing.^9^ Studies in Indian hospitals have found that 68% of MDR Gram-negative bacteria^12^ and 62.5% colistin-resistant Gram-negative bacteria^13^ were susceptible to chloramphenicol. In Malawi chloramphenicol was used in combination with benzylpenicillin as the first-line therapy in the empirical management of suspected sepsis, until 2015.^4^ Irrespective of the national guidelines, ceftriaxone has been widely used in Malawi since 2005, partly due to ease of administration (once daily) and partly because the most common cause of bloodstream infection was MDR *Salmonella enterica*,^14^ reducing chloramphenicol use. Susceptibility to chloramphenicol has been gradually returning; resistance rates in 2016 were 61% in *Escherichia coli* and 48% in *Klebsiella pneumoniae* compared with 80% of *E. coli*^15^ and 81% of *K. pneumoniae* in 2001.^4^ The regulated reintroduction of chloramphenicol for the treatment of susceptible isolates could reduce the duration of courses of third-generation cephalosporins, protecting these vital agents.^8^

Chloramphenicol resistance is most commonly due to enzymatic inactivation by chloramphenicol acetyltransferases (CAT), of which there are two structurally distinct types: CAT-A and CAT-B.^16^ There are at least 16 *catA* genes, with *catA1–3* found in Enterobacteriaceae,^16–18^ alongside *catB2* and *catB3* group 3 (*catB3–6* and *catB8*).^16^ Resistance also occurs due to efflux mechanisms mediated by *cml* and *floR* genes.^16^ Additional chloramphenicol resistance genes found in Gram-positive species include *fexA* and *fexB* from *Staphylococcus*, the ABC-F gene *optrA* from *Enterococcus* spp.^19^ and *Staphylococcus* spp.,^20^ and the multiresistance genes *cfr*, *cfr*(B) and *cfr*(C), which have been sporadically detected in *Proteus* spp. and *E. coli*.^21^

Typically, phenotypic testing requires an overnight culture step to isolate the organism, followed by additional culture-based drug susceptibility testing with results available within 48–72 h. This delay results in the prolonged use of empirical broad-spectrum antimicrobials and drives resistance. Molecular assays, however, can be performed from the initial overnight culture, with a result available within 2–3 h, including the DNA extraction process. We report here the design of an RT-PCR assay to detect chloramphenicol susceptibility in *E. coli* and *Klebsiella* spp. employing high-resolution melt (HRM) analysis, which enables a high degree of multiplexing, without expensive fluorescent probes.^22^

## Materials and methods

### Ethics

The collection of the isolates during routine surveillance was approved by the University of Malawi College of Medicine Research and Ethics Committee (COMREC), Blantyre, under study number P.08/14/1614.

### Bacterial isolates

Seventy-two previously whole-genome sequenced bacterial isolates,^15^ all from separate patients, were collected in Queen Elizabeth Central Hospital, Blantyre, Malawi between 1998 and 2016. The collection consisted of 39 *E. coli* and 33 *K. pneumoniae*, and included 61 isolated from blood culture, 10 from CSF and 1 from a rectal swab.

Isolates were transported to the Liverpool School of Tropical Medicine (LSTM), Liverpool, UK on microbiological beads (Biobank) on dry ice where they were stored at −80°C.

### Identifying resistance genes from WGS data

The sequencing of the isolates using the Illumina Hiseq 2000 platform (Illumina, Inc., San Diego, CA, USA), de novo assembly and sequence annotation was reported by Musicha *et al.*^15^

The final annotated sequences were screened for acquired AMR genes using NCBI BLAST against a bespoke database of genes curated by the Wellcome Trust Sanger Institute based on the ResFinder database. A cut-off of >95% for nucleotide identity and >90% for coverage against the database was used to confirm gene identity.

### DNA extraction

Cultures were revived from microbiological beads (BioBank) using Mueller–Hinton agar (Sigma) plates and incubated at 37°C overnight. Single colonies were selected and DNA was extracted using a DNeasy Blood and Tissue kit (Qiagen, Germany) following the protocol for Gram-negative bacteria.

### Chloramphenicol MICs

Chloramphenicol MIC assays were performed following EUCAST 2018 guidelines.^23^ Briefly, a single colony from each isolate was added to 5 mL of Mueller–Hinton broth and incubated at 37°C overnight. The culture was diluted to 1 × 10^5^ cfu/mL and added to a 96-well plate containing triplicates of 512 to 0.2 mg/L chloramphenicol in Mueller–Hinton broth and incubated at 37°C overnight. A concentration of 8 mg/L was used as the breakpoint for resistance as per the EUCAST guidelines.^23^

### Primer design

Primers were designed for all the major chloramphenicol resistance genes found in Enterobacteriaceae: *catA1*, *catA2*, *catA3*, *catB2*, *catB3* group 3 (including *catB3*, *catB4*, *catB5*, *catB6* and *catB8*), *cmlA* (*cml*, *cmlA*, *cmlA1*, *cmlA3*, *cmlA4*, *cmlA5*, *cmlA6* and *cmlA7*) and *floR*.^16^^,^^24^ Sequences for each gene were downloaded from NCBI GenBank and aligned using ClustalX in MEGA 7.0.14 to identify conserved sites.^15^ The 16S rRNA primers were designed with a mismatch on the 3′ end of the forward primer to any non-Enterobacteriaceae species preventing amplification,^25^ and produced separate peaks for *K. pneumoniae* and *E. coli*. Primers were designed using Primer3 (http://primer3.ut.ee/) to give each amplicon a distinct melting temperature (Tm) for identification following HRM analysis. Amplicon Tm was estimated using the nearest neighbour method on OligoCalc (http://biotools.nubic.northwestern.edu/OligoCalc.html). Primer sequences, concentrations and amplicon details are displayed in Table S1 (available as Supplementary data at *JAC* Online).

**Table 1.** Accuracy of the ChloS-HRM assay compared with MIC results and the associated treatment outcomes for each isolate

	True susceptible (correctly de-escalated to chloramphenicol)	True resistant (correctly continue β-lactam therapy)	False susceptible (incorrect de-escalation: potential treatment failure)	False resistant (incorrectly continue β-lactam therapy: overuse)
*E. coli*	12	25	1	1
*K. pneumoniae*	3	29	0	1
Total	15/17 (88%)	54/55 (98%)	1/55 (2%)	2/17 (12%)

### ChloS-HRM assay

The ChloS-HRM assay was performed in a total volume of 12.5 μL, consisting of 6.25 μL of 2× HRM Type-It mix (Qiagen), variable concentrations of each primer (Table S1), molecular-grade water and 2.5 μL of the DNA template. All HRM development and evaluation runs were performed on a Rotor-Gene Q 6000 (Qiagen, Germany) with the following conditions: Taq activation at 95°C for 5 min, then 30 cycles of 95°C for 10 s, 62°C for 30 s and 72°C for 20 s. The subsequent HRM step consisted of melting from 73°C to 89°C, reading at every 0.1°C step with a 2 s stabilization. Data analysis was carried out using the Rotor-Gene Q software.

Isolates carrying chloramphenicol resistance genes according to WGS data were used to confirm the Tm and location of the peak for each gene. *catA3* and *catB* were not available in the Malawian isolates and therefore synthesized sequences of *catA3* (NCBI reference sequence: NG_052661.1) and *catB2* (NCBI reference sequence: NG_047602.1) (Eurofins) and a *K. pneumoniae* isolate from the LSTM archive carrying the *catB3* gene were used as positive controls. Calling bins were set for each gene, including 16S rRNA, and a positivity cut-off of 10% maximum peak fluorescence was set at 0.5 d*F*/d*T* (Figure S1). Any isolate with a peak within a defined calling bin over the positivity cut-off value was classified as positive for the corresponding gene. Any isolate that generated no resistance peaks but produced a 16S rRNA peak was classified as susceptible to chloramphenicol.

### Pilot study

The 72 bacterial isolates were tested with the ChloS-HRM assay using the pre-defined calling bins and positivity cut-off value by an operator blinded to the MIC and sequencing results. The ChloS-HRM results were compared against the MIC results as a reference standard and the detection of specific genes was compared with WGS data.

### Hypothetical cohorts

To estimate the performance of ChloS-HRM we determined assay outcomes with hypothetical cohorts of 1000 patients in areas with differing levels of chloramphenicol resistance prevalence (90% to 10%). The negative predictive value (NPV), positive predictive value (PPV) and an estimation of the number of cases where treatment could change were calculated.

## Results

### MICs

**Figure 1.**
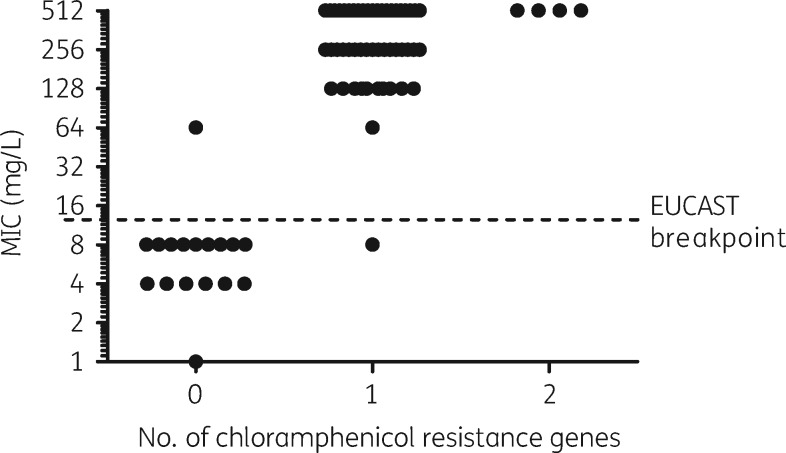
MIC data for the 72 isolates showing the effect of chloramphenicol resistance gene carriage as characterized by WGS.

A total of 55 isolates, 26 *E. coli* and 29 *K. pneumoniae*, were classified as resistant, with an MIC range of 64–512 mg/L, and 17 isolates, 13 *E. coli* and 4 *K. pneumoniae*, were classified as susceptible, with an MIC range of 1–8 mg/L (Figure 1).

### Comparison of chloramphenicol resistance gene carriage by WGS and MIC

Of the 17 susceptible isolates, 1 contained a *floR* gene by WGS and had an MIC of 8 mg/L giving WGS an accuracy of 94% (95% CI: 71.31%–99.85%) in detecting susceptibility. All other susceptible isolates had no resistance genes by WGS. Of the 55 resistant isolates, 50 had a single resistance gene by WGS; 49 contained *catA* and 1 isolate contained *floR*. Four isolates had two resistance genes (2× *catA* and *cmlA*, 1× *catA* and *floR*, and 1× *cmlA* and *floR*), all of which had an MIC of 512 mg/L (Figure 1). A single isolate with an MIC of 64 mg/L had no AMR gene by WGS, giving WGS an accuracy of 98% (95% CI: 90.28%–99.95%) in identifying resistant isolates.

### Pilot study

The ChloS-HRM results were compared with MIC data to determine its accuracy in identifying susceptible isolates (Table 1). The HRM assay correctly identified 15 of 17 chloramphenicol-susceptible isolates, giving an accuracy of 88% to identify susceptibility (95% CI: 63.56%–98.54%). Of the two isolates falsely classified as resistant by ChloS-HRM, one had a peak for *catB* group 3 and the other had a peak for *floR*; this isolate was also positive for *floR* by WGS. A total of 54 of 55 MIC resistant isolates were classified correctly by the HRM assay, giving an accuracy of 98% (95% CI: 90.28%–99.95%) in identifying resistant isolates. The single false-susceptible result had an MIC of 64 mg/L but did not have any resistance genes detected by HRM or WGS.

Compared with the WGS data the ChloS-HRM assay had 100% sensitivity and 100% specificity for detecting *catA* (including *catA1–3*), *cmlA* and *floR* (Table 2). The assay had a specificity of 96% for *catB* group 3; we were unable to estimate sensitivity due to the lack of this gene in the sample collection. All four isolates with two resistance genes by WGS were correctly identified by ChloS-HRM.

**Table 2.** Sensitivity and specificity of the ChloS-HRM assay in detecting resistance markers compared with WGS

	HRM versus WGS			
	*catA*	*catB*	*cmlA*	*floR*
Sensitivity (%)	100 (52/52 TP)	–	100 (3/3 TP)	100 (4/4 TP)
Specificity (%)	100 (20/20 TN)	96 (70/72 TN)	100 (69/69 TN)	100 (68/68 TN)

TP, true positive; TN, true negative.

The overall agreement between MIC testing and HRM was 96% in classifying isolates, while the overall agreement between MIC testing and WGS was 97%. See Table S2 for full MIC, WGS and ChloS-HRM data for each isolate.

A 16S peak was detected in 65 of the 72 isolates (90%); all 7 isolates without a 16S peak had at least one resistance marker peak, indicating a successful RT-PCR. A total of 16 of 16 HRM susceptible isolates produced a 16S peak and the correct species was identified in 64 of 64 isolates (100%).

### Hypothetical cohorts

The hypothetical cohort indicated that as susceptibility prevalence increases, so too does the PPV in identifying susceptibility, while the number of false-susceptible cases decreases (Table 3). Thus, the higher the prevalence of susceptibility, the more effective the assay is. Once susceptibility drops <30%, where the PPV and NPV are both 95%, there are too many false-susceptible results for the assay to be effective. The most recent data in Malawi from 2016 indicated a susceptibility prevalence of 39% in *E. coli* giving a PPV of 97% and an NPV of 93%, and a susceptibility prevalence of 52% in *K. pneumoniae* giving a PPV of 98% and an NPV of 89%.^4^

**Table 3.** Test outcomes in hypothetical cohorts of 1000 bacteraemia cases with variable prevalence of chloramphenicol susceptibility

Susceptibility prevalence (%)	No. of susceptible infections	No. of resistant infections	True susceptible (correctly use chloramphenicol: treatment success)	True resistant (correctly use ceftriaxone: treatment success)	False susceptible (incorrectly use chloramphenicol: treatment failure)	False resistant (incorrectly use ceftriaxone: treatment success, but drug overuse)	PPV: correctly identifying susceptibility	NPV: correctly identifying resistance
10	100	900	88	882	18	12	83.0	98.7
20	200	800	176	784	16	24	91.7	97.0
30	300	700	265	686	14	35	95.0	95.1
40	400	600	353	588	12	47	96.7	92.6
50	500	500	441	490	10	59	97.8	89.3
60	600	400	529	392	8	71	98.5	84.7
70	700	300	617	294	6	83	99.0	78.0
80	800	200	706	196	4	94	99.4	67.6

## Discussion

We have developed a highly multiplexed HRM RT-PCR assay capable of detecting 18 different chloramphenicol resistance genes within seven melt peaks, along with a bacterial 16S rRNA control enabling discrimination of *E. coli* and *K. pneumoniae*. These are the two most prevalent causes of Gram-negative bacteraemia, excluding *Salmonella* spp., in Malawi, and the pathogens most strongly associated with ESBL production. The assay was used to detect chloramphenicol-susceptible isolates in Malawi, where rapid de-escalation from broad-spectrum β-lactams such as ceftriaxone could be possible.

The assay was 88% accurate in identifying phenotypically susceptible isolates and 98% accurate in identifying resistant isolates. Accurately identifying resistant infections is vital; false-susceptible results would lead to the incorrect use of chloramphenicol on a resistant infection, likely leading to treatment failure. The lower accuracy in detecting susceptible infections would have less impact on treatment success, as a false-resistant result would lead to the continuation of broad-spectrum empirical therapy.

Molecular testing of AMR genes has been shown to correlate well with phenotypic resistance in Enterobacteriaceae,^26^^,^^27^ and our comparison of WGS data with MIC testing showed a 97% agreement. However, as PCR-based methods detect the presence, but not expression level or copy number of a gene, the effect of these changes of transcription levels is not reflected in the result.^28^ PCR methods also cannot distinguish silenced resistance genes, in these cases predicting resistance in susceptible isolates. Silent resistance genes are sometimes reactivated in response to antibiotic pressure but often remain silent.^29^

All *catA*, *cmlA* and *floR* genes were correctly detected; however, the degenerate *catB* group 3 primers produced 3 of 72 false-positive results; two of these isolates contained other resistance genes and so did not affect the overall result. Degenerate primers enable the detection of multiple *catB* genes, maximizing sensitivity, but can lead to a higher likelihood of non-specific binding.^30^ In the two isolates containing only the *floR* resistance gene, one had an MIC of 64 mg/L and the other an MIC of 8 mg/L, making it chloramphenicol susceptible. WGS analysis did not reveal any SNPs in the gene from this isolate^15^; however, the *floR* gene has previously been shown to confer variable resistance.^31^^,^^32^

All chloramphenicol resistance genes reported in the Enterobacteriaceae were included in the assay, with some rarely encountered exceptions.^16^^,^^24^ All genes identified in Malawi during a previous project sequencing resistant invasive isolates were included.^15^ The MDR gene *cfr*, found predominantly in Gram-positive species, but also rarely reported in *E. coli* and *Proteus*,^21^ was excluded. Data on *cfr* in *E. coli* and *K. pneumoniae* are still very scarce, particularly in sub-Saharan Africa.

The Enterobacteriaceae 16S internal control was successfully amplified in 65 of 72 (90%) isolates. 16S primer concentrations were deliberately lower than the other sets to prevent amplification of the 16S gene occurring to the detriment of more important resistance genes. All seven of the samples where the peak was absent contained at least one resistance peak.

Estimation of test outcomes on hypothetical cohorts illustrated that as the prevalence of susceptibility increases, the number of false-susceptible infections decreases, thus increasing the PPV and enhancing the potential for de-escalation of therapy. Chloramphenicol resistance in the Enterobacteriaceae ranges between 31% and 94.2% depending on the setting;^3^^,^^12^ therefore, the implementation of this test must be considered alongside local resistance rates.

Recent WGS studies in Malawi found that 56 of 134 (41.8%) of ESBL-containing *E. coli*^15^ and 41 of 262 (15.6%) ESBL-containing *K. pneumoniae*^39^ were chloramphenicol susceptible, highlighting that the ChloS-HRM assay would prove effective in de-escalating to a more effective treatment option, particularly in *E. coli*. Furthermore, reports of chloramphenicol-susceptible *S. enterica* are also increasing.^33–37^ As resistance is mediated by the genes that the ChloS-HRM assay identifies,^16^ it could potentially identify susceptibility in this species. The identification of chloramphenicol susceptibility could also provide a welcome addition to the diminishing options available for treating for MDR and XDR Enterobacteriaceae.

Affordability of antibiotics is critical for low- and middle-income countries, particularly in countries such as Malawi where the public healthcare system provides treatment for free.^38^ Chloramphenicol is highly affordable in comparison with carbapenems, the next line of antimicrobial chemotherapy, and increased use would likely be cost saving.

The ChloS-HRM assay enables the sensitive detection of chloramphenicol-susceptible *E. coli* and *K. pneumoniae*, allowing the de-escalation to chloramphenicol and enhancing antimicrobial stewardship. De-escalation to chloramphenicol would also prevent escalation to carbapenems and so would also be protective of this class of drugs as well as cephalosporins. The test would be a powerful tool for molecular epidemiology and surveillance studies on chloramphenicol resistance, where WGS is not feasible.

## Supplementary Material

Supplementary Data
