# Supplementary material for: ChloS-HRM, a novel assay to identify chloramphenicol-susceptible Escherichia coli and Klebsiella pneumoniae in Malawi
Source: J Antimicrob Chemother. 2019 Jan 25;74(5):1212–7. doi: 10.1093/jac/dky563 (PMC6477986; doi:10.1093/jac/dky563)
Supplement: Supplementary Data [file dky563_supplementary_data.docx]

**Supplementary data**

**Table S1.** Primer sequences, concentrations and amplicon melt temperatures for the ChloS-HRM assay

| Target | Forward Primer (5'-3') | Reverse Primer (5'-3') | Primer Concentration (nM) | Amplicon Size (bp) | Amplicon Tm (°C) |
| --- | --- | --- | --- | --- | --- |
| *CatA1* | AATAAGATCACTACCGGGCGT | GCAACTGACTGAAATGCCTCA | 400 | 150 | 78.43 |
| *CatA2* | ACCCATTGAGACAACCAGACT | CGATTCCAGGTATTCAGGTCAA | 600 | 106 | 74.6 |
| *CatA3* | CGGCATCGTTTACCATGTG | TCAAATTGATTCACGGCCTGA | 400 | 137 | 77.4 |
| 16S – internal control | TACACACGTGCTACAATGGC | TCATGGAGTCGAGTTGCAGA | 100 | 128 | 81.06 (Kp), 82.77 (Ec) |
| CatB2 | AGGAAGCGCTTTTCTGAAGAA | GCAATGCCAGACGAACAAAGA | 400 | 113 | 79.11 |
| *CatB* Group 3 including; *CatB3*, *CatB4*, *CatB5*, *CatB6*, *CatB8* | GACTGGGYAWCATCYTTCCC | CACCAACGAKCGRCTRCCT | 700 | 185 | 83.88 |
| *cmlA* including; *cml*, *cmlA* *cmlA1*, *cmlA3*, *cmlA4*, *cmlA5*, *cmlA6*, *cmlA7* | ACGGCATACTCGGATCCATG | CTTAACGGGGAGTAGCAGCT | 300 | 212 | 87.58 |
| *floR* | ATGGCTCCTTTCGACATCCT | CAAGTAGAATTGGCCGTCGC | 400 | 196 | 85.2 |

**Table S2.** MIC, WGS and ChloS-HRM data for each of the 72 isolates

| **Isolate** | **Source** | **MIC (mg/L)** | **MIC Susceptible** | **No. Resistant Genes - WGS** | **Gene(s) - WGS** | **WGS Susceptible** | **No. Resistant Genes - HRM** | **Genes - HRM** | **HRM Susceptible** |
| --- | --- | --- | --- | --- | --- | --- | --- | --- | --- |
| Ec 1010805 | Blood | 4 | 1 | 0 |  | 1 | 0 |  | 1 |
| Ec 10129 | CSF | 8 | 1 | 0 |  | 1 | 0 |  | 1 |
| Ec 10140 | CSF | 128 | 0 | 1 | CatA | 0 | 1 | CatA1 | 0 |
| Ec 1014142 | Blood | 512 | 0 | 1 | CatA | 0 | 1 | CatA1 | 0 |
| Ec 10151 | CSF | 256 | 0 | 1 | CatA | 0 | 1 | CatA1 | 0 |
| Ec 1016948 | CSF | 4 | 1 | 0 |  | 1 | 0 |  | 1 |
| Ec 2209 | Blood | 512 | 0 | 1 | CatA | 0 | 1 | CatA1 | 0 |
| Ec 2473 | Blood | 4 | 1 | 0 |  | 1 | 0 |  | 1 |
| Ec 2558 | Blood | 256 | 0 | 1 | CatA | 0 | 1 | CatA1 | 0 |
| Ec 36329 | Blood | 256 | 0 | 1 | CatA | 0 | 1 | CatA1 | 0 |
| Ec 4464 | CSF | 1 | 1 | 0 |  | 1 | 0 |  | 1 |
| Ec 4600 | Blood | 256 | 0 | 1 | CatA | 0 | 1 | CatA1 | 0 |
| Ec 8728 | CSF | 4 | 1 | 0 |  | 1 | 0 |  | 1 |
| Ec 9693 | Blood | 128 | 0 | 1 | CatA | 0 | 1 | CatA1 | 0 |
| Ec A38084 | Blood | 8 | 1 | 0 |  | 1 | 1 | CatB3 | 0 |
| Ec A38988 | Blood | 128 | 0 | 1 | CatA | 0 | 1 | CatA1 | 0 |
| Ec A40286 | Blood | 64 | 0 | 0 |  | 1 | 0 |  | 1 |
| Ec A45016 | Blood | 8 | 1 | 0 |  | 1 | 0 |  | 1 |
| Ec A48349 | Blood | 8 | 1 | 0 |  | 1 | 0 |  | 1 |
| Ec A5175 | Blood | 256 | 0 | 1 | CatA | 0 | 1 | CatA1 | 0 |
| Ec A7503 | Blood | 128 | 0 | 1 | CatA | 0 | 1 | CatA1 | 0 |
| Ec AB6140 | Blood | 4 | 1 | 0 |  | 1 | 0 |  | 1 |
| Ec B9070 | CSF | 8 | 1 | 0 |  | 1 | 0 |  | 1 |
| Ec BK07M9 | Blood | 8 | 1 | 0 |  | 1 | 0 |  | 1 |
| Ec C12359 | CSF | 128 | 0 | 1 | CatA | 0 | 1 | CatA1 | 0 |
| Ec D25640 | Blood | 512 | 0 | 1 | CatA | 0 | 1 | CatA1 | 0 |
| Ec D25641 | Blood | 256 | 0 | 1 | CatA | 0 | 1 | CatA1 | 0 |
| Ec D32322 | Blood | 512 | 0 | 1 | CatA | 0 | 2 | CatA1, CatA2 | 0 |
| Ec D3420 | Blood | 128 | 0 | 1 | CatA | 0 | 1 | CatA1 | 0 |
| Ec D3475 | Blood | 8 | 1 | 0 |  | 1 | 0 |  | 1 |
| Ec D37334 | Blood | 128 | 0 | 1 | CatA | 0 | 1 | CatA1 | 0 |
| Ec D3787 | Blood | 512 | 0 | 2 | CatA, flor | 0 | 2 | CatA1, floR | 0 |
| Ec D39719 | Blood | 512 | 0 | 1 | CatA | 0 | 2 | CatA1, CatA2 | 0 |
| Ec D40034 | Blood | 512 | 0 | 1 | CatA | 0 | 1 | CatA1 | 0 |
| Ec D42544 | Blood | 256 | 0 | 1 | CatA | 0 | 1 | CatA1 | 0 |
| Ec D4531 | Blood | 128 | 0 | 1 | CatA | 0 | 1 | CatA1 | 0 |
| Ec D46760 | Blood | 512 | 0 | 1 | CatA | 0 | 2 | CatA1, CatA2 | 0 |
| Ec D48799 | Blood | 128 | 0 | 1 | CatA | 0 | 1 | CatA1 | 0 |
| Ec D49086 | Blood | 256 | 0 | 1 | CatA | 0 | 1 | CatA1 | 0 |
| Kl 1007011 | Blood | 256 | 0 | 1 | CatA | 0 | 2 | CatA1, CatA2, CatB3 | 0 |
| Kl 1011136 | Blood | 8 | 1 | 0 |  | 1 | 0 |  | 1 |
| Kl 1022430 | Blood | 512 | 0 | 2 | CatA, cmlA | 0 | 1 | CatA2, cmlA | 0 |
| Kl 1023547 | Blood | 512 | 0 | 1 | CatA | 0 | 1 | CatA2 | 0 |
| Kl 1027734 | Blood | 512 | 0 | 2 | CatA, cmlA | 0 | 3 | CatA1, CatA2, cmlA | 0 |
| Kl 3208 | Blood | 512 | 0 | 1 | CatA | 0 | 1 | CatA2 | 0 |
| Kl 3712 | Blood | 256 | 0 | 1 | CatA | 0 | 1 | CatA1 | 0 |
| Kl 7776 | Blood | 8 | 1 | 1 | floR | 0 | 1 | floR | 0 |
| Kl 8407 | Blood | 512 | 0 | 1 | CatA | 0 | 1 | CatA2 | 0 |
| Kl A44574 | Blood | 256 | 0 | 1 | CatA | 0 | 1 | CatA1 | 0 |
| Kl A45155 | Blood | 512 | 0 | 1 | CatA | 0 | 1 | CatA2 | 0 |
| Kl B1040 | CSF | 8 | 1 | 0 |  | 1 | 0 |  | 1 |
| Kl BKQ 2KD | Blood | 512 | 0 | 1 | CatA | 0 | 1 | CatA2 | 0 |
| Kl BKQ 2QU | Blood | 256 | 0 | 1 | CatA | 0 | 1 | CatA2 | 0 |
| Kl D25446 | Blood | 512 | 0 | 1 | CatA | 0 | 1 | CatA2 | 0 |
| Kl D25597 | Blood | 512 | 0 | 1 | CatA | 0 | 1 | CatA1 | 0 |
| Kl D29665 | Blood | 512 | 0 | 1 | CatA | 0 | 1 | CatA1 | 0 |
| Kl D35263 | Blood | 64 | 0 | 1 | floR | 0 | 1 | floR | 0 |
| Kl D37700 | Blood | 512 | 0 | 1 | CatA | 0 | 1 | CatA2 | 0 |
| Kl D39172 | Blood | 512 | 0 | 2 | cmlA, floR | 0 | 2 | cmlA, floR | 0 |
| Kl D40180 | Blood | 512 | 0 | 1 | CatA | 0 | 1 | CatA2 | 0 |
| Kl D40443 | Blood | 512 | 0 | 1 | CatA | 0 | 1 | CatA2 | 0 |
| Kl D44912 | Blood | 256 | 0 | 1 | CatA | 0 | 1 | CatA2 | 0 |
| Kl D46208 | Blood | 256 | 0 | 1 | CatA | 0 | 1 | CatA2 | 0 |
| Kl D47091 | Blood | 256 | 0 | 1 | CatA | 0 | 2 | CatA1, CatA2 | 0 |
| Kl D4743 | Blood | 4 | 1 | 0 |  | 1 | 0 |  | 1 |
| Kl D4888 | Blood | 128 | 0 | 1 | CatA | 0 | 1 | CatA2 | 0 |
| Kl D49363 | Blood | 512 | 0 | 1 | CatA | 0 | 3 | CatA1, CatA2, CatB3 | 0 |
| Kl D50395 | Blood | 512 | 0 | 1 | CatA | 0 | 2 | CatA1, CatA2 | 0 |
| Kl D5105 | Blood | 512 | 0 | 1 | CatA | 0 | 1 | CatA1 | 0 |
| Kl D51871 | Blood | 512 | 0 | 1 | CatA | 0 | 1 | CatA2 | 0 |
| Kl D53369 | Blood | 256 | 0 | 1 | CatA | 0 | 2 | CatA1, CatA2 | 0 |
| Kl O1408 | Rectal Swab | 256 | 0 | 1 | CatA | 0 | 1 | CatA2 | 0 |


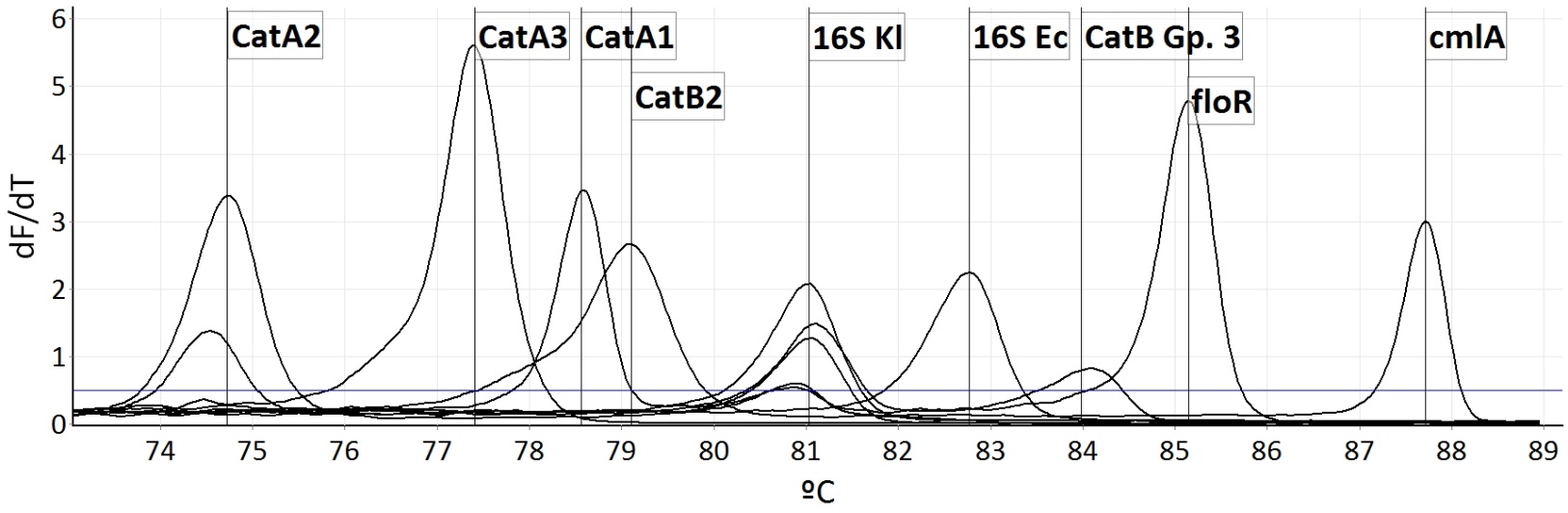


**Figure S1.** Melt curve profile of the genes targeted by the assay. Multiple peaks are present for *CatA2* due to co-carriage of gene by the isolate with *cmIA*. The detection threshold is indicated by the horizontal blue line, and automatic calling bins by the vertical grey lines.
